# Study of a High-Yield Cellulase System Created by Heavy-Ion Irradiation-Induced Mutagenesis of *Aspergillus niger* and Mixed Fermentation with *Trichoderma reesei*

**DOI:** 10.1371/journal.pone.0144233

**Published:** 2015-12-11

**Authors:** Shu-Yang Wang, Bo-Ling Jiang, Xiang Zhou, Ji-Hong Chen, Wen-Jian Li, Jing Liu, Wei Hu, Guo-Qing Xiao, Miao-Yin Dong, Yu-Chen Wang

**Affiliations:** 1 Institute of Modern Physics, Chinese Academy of Sciences, 509 Nanchang Rd, Lanzhou, Gansu 730000, PR China; 2 Lanzhou University, 222 South Tianshui Road, Lanzhou, Gansu 730000, PR China; 3 University of Chinese Academy of Sciences, No.19A Yuquan Road, Beijing 100049, PR China; National Renewable Energy Lab, UNITED STATES

## Abstract

The aim of this study was to evaluate and validate the efficiency of ^12^C^6+^ irradiation of *Aspergillus niger* (*A*. *niger*) or mutagenesis via mixed *Trichoderma viride* (*T*. *viride*) culturing as well as a liquid cultivation method for cellulase production via mixed *Trichoderma reesei* (*T*. *reesei*) *and A*. *niger* culture fermentation. The first mutagenesis approach was employed to optimize yield from a cellulase-producing strain via heavy-ion mutagenesis and high-throughput screening, and the second was to effectively achieve enzymatic hydrolysis of cellulase from a mixed culture of mutant *T*. *viride* and *A*. *niger*. We found that ^12^C^6+^-ion irradiation induced changes in cellulase biosynthesis in *A*. *niger* but had no effect on the time course of the synthesis. It is notable that the exoglucanases (CBH) activities of *A*. *niger* strains H11-1 and H differed (6.71 U/mL vs. 6.01 U/mL) and were significantly higher than that of *A*. *niger* mutant H3-1. Compared with strain H, the filter paper assay (FPA), endoglucanase (EG) and *β*-glucosidase (BGL) activities of mutant strain H11-1 were increased by 250.26%, 30.26% and 34.91%, respectively. A mixed culture system was successfully optimized, and the best ratio of *T*. *reesei* to *A*. *niger* was 5:1 for 96 h with simultaneous inoculation. The BGL activity of the mixed culture increased after 72 h. At 96 h, the FPA and BGL activities of the mixed culture were 689.00 and 797.15 U/mL, respectively, significantly higher than those of monocultures, which were 408.70 and 646.98 U/mL for *T*. *reesei* and 447.29 and 658.89 U/mL for *A*. *niger*, respectively. The EG activity of the mixed culture was 2342.81 U/mL, a value that was significantly higher than that of monocultures at 2206.57 U/mL for *T*. *reesei* and 1727.62 U/mL for *A*. *niger*. In summary, cellulose production and hydrolysis yields were significantly enhanced by the proposed combination scheme.

## Introduction

There is increasing interest in sustainable methods to meet the growing energy demands of transportation, heating and industrial processes and in providing raw materials for industry [1]. However, as the use of food as a raw material for biological and industrial fuels has caused global concerns of food safety and significantly affected the public acceptance of biofuels, biofuel research is focusing on the development of next-generation biofuels. To this end, the synthesis of bioethanol from lignocellulosic raw materials, e.g., agricultural and forestry residues, portions of municipal waste, and herbaceous and woody crops, is a promising option for non-food biofuel production [2,3]. Although bioethanol is widely recognized as a unique transportation fuel with powerful economic, environmental and strategic attributes [4], fermentation of lignocellulosic biomass to ethanol is still in the initial stages of development, and some technological processes require further improvement.

The effective conversion of recalcitrant lignocellulose into fermentable sugars is a key challenge in lignocellulosic bioethanol production. In nature, many carbohydrate-active enzymes are involved in breaking down cellulolytic biomass, including glycosyltransferases and polysaccharide lyases. However, in industrial applications, a pre-treatment process is typically required, followed by enzymatic hydrolysis catalyzed by cellulases and other glycosyl hydrolases (GHs) [5,6]. Therefore, leveraging new technologies to reduce costs is essential for bioethanol industrialization. Indeed, cellulases are still comparatively costly, and the need to reduce production costs is of great importance for their industrial and commercial use in biorefineries [7].

Cellulose production costs may be decreased via multifaceted approaches, including the use of cheap lignocellulosic substrates and cost-efficient fermentation strategies, e.g., solid-state fermentation [8]. Microbes with high growth rates such as *Trichoderma viride* have the potential to surpass slower growing fungi in cost-effective cellulase production. Indeed, *T*. *viride* is one of the most crucial microorganisms used in industry because it exhibits a higher enzyme yield with cellulose, as measured by the filter paper assay (FPA), which reflects the magnitude of its cellulose-decomposing ability [9]. Cellulase activity consists of three components: β-glucosidases (BGLs), exoglucanases (CBHs) and endoglucanases (EGs). Cellobiohydrolases are CBHs that release cellobiose as the main product from crystalline cellulose. EGs preferably attack amorphous cellulose and some short-chain oligomers, whereas BGLs hydrolyze cello-oligosaccharides and cellobiose into glucose [10–12]. *In vivo* biological production systems utilizing *Trichoderma reesei* are deficient in BGL activity, which leads to the accumulation of cellobiose and subsequent product inhibition of endo- and exo-glucanases [13]. However, the inhibitory effects of cellobiose accumulation on enzymatic hydrolysis can be diminished by adding external BGL [14,15] or employing mixed fermentation with *Aspergillus niger* [16,17], the cellulase system of which is rich in BGL and effectively decomposes cellulose (cellobiose) into two glucose molecules, reducing the end-product inhibition of this enzyme and its expression.

One of the most important and convenient physical methods for obtaining broad-spectrum mutations is heavy-ion radiation; such beams improve the mutation rate, broaden the mutation spectrum and shorten the breeding cycle [18,19]. In fact, heavy ion-based microbial breeding methods readily result in mutants with high mutation frequencies and genetic stability and are powerful tools for generating mutant libraries [20–22].

Previous studies have described various aspects of cellulose production. However, a systematic study comparing the efficiency of *A*. *niger* mutagenesis using ^12^C^6+^ irradiation versus mixed culturing with *T*. *viride* has not been conducted. Moreover, there are no reports concerning cellulase production using the heavy ion-induced mutation technique and mixed-strain fermentation. Therefore, the aim of the present work was to evaluate and validate the efficiency of *A*. *niger* mutagenesis with ^12^C^6+^ irradiation or mixed culturing with *T*. *viride* and the subsequent performance of the mutant *A*. *niger* in a novel sequential liquid cultivation method for cellulase production via mixed culture fermentation with *T*. *reesei*. This technique was based on the breeding of high-yield β-glucosidase mutants of *A*. *niger* using a carbon ion-induced mutation approach. Ultimately, a mixed culture system of mutant *T*. *reesei* NMy and *A*. *niger* H-11 was selected to yield cellulase via the greatest enzymatic activities.

## Materials and Methods

### Heavy-ion beam irradiation mutagenesis

#### Experimental setup

Heavy-ion beam experimental setups were employed as previously described [23]. Briefly, the extraction time of the carbon ions (approximately 10^6^−10^8^ ions/pulse) was approximately 3 s, and the priming dose was 0–140 Gy. The dose rates were up to 20 Gy min^-1^.

#### Heavy-ion beam irradiation

*A*. *nige*r was cultured on different substrates, such as potato dextrose agar (PDA) slants. For chemostat cultures grown at 30°C, once good sporulation occurred after 5 days, the cultures were inoculated into triangular shake flasks with glass beads and cultured for 3 h in a rotary shaker (240 rpm) at 30°C. Then, 3 mL of a single-spore suspension was transferred into an irradiated dish (USA, size 35×10 mm) for each sample as an irradiation target. The spore suspensions were exposed to ^12^C^6+^ ion beams (at 80 AMeV) for a priming dose of 20–140 Gy. Three parallel groups for each sample were irradiated at each dose. Each of the irradiated spore suspensions was 10-fold serially diluted; a cellulose-Congo Red separation plate was coated with a 1:10,000 dilution, and the culture was grown for 60 hours in an incubator at 30°C.

After irradiating the inoculated plates, the lethality and mutation rates were calculated using the following equations:

Lethalityrate=[T/U]×100% (1)

where T is the number of colonies after heavy-ion beam irradiation treatment and U is the number of colonies without heavy-ion irradiation treatment.

Positionmutationrate=[M1/T]×100% (2)

where M1 is the total number of colonies of the positive mutant strain and T is the number of colonies after heavy-ion beam irradiation. A growth circle-to-colony (HC) ratio greater than 1.80 on cellulose-Congo Red screening plates indicates a positive mutation.

Negativemutationrate=[M2/T]×100% (3)

where M2 is the total colony forming units of the mutant strain and T is the number of colonies after heavy-ion beam irradiation. An HC ratio less than 1.40 on cellulose-Congo Red indicates a negative mutation.

### Breeding of mutant strains

Single colonies with HC ratios greater than 1.8 were inoculated into flasks that had been prepared by autoclaving the following mixture at 121°C for 15 min: 40 g/L wheat bran, 20 g/L Avicel, 50 g/L glucose, 0.16 g/L of MgSO_4_, 0.5 g/L of Fe_2_(SO_4_)_3_, 2 g/L (NH_4_)_2_SO_4_, 0.2 g/L NaCl and 0.5 g/L of KH_2_PO_4_. The shake flasks were subsequently incubated in a shaking water bath at 30°C and 300 rpm for 3 days. The fermentation liquid was diluted 10-fold, and 1.0 mL was added to 15-mL test tubes to which 2.0 mL of DNS reagent was added; the mixture was then boiled for 2 min. After cooling, water was added to 15 mL. The OD values were determined at a wavelength of 540 nm. The reducing sugar concentration was determined by the DNS method [24].

### Microorganisms

*A*. *niger* GSTCC 60108 (NH01) and *T*. *viride* GSTCC 62010 (NM01) were obtained from the industrial microbial culture collection center of Gansu Province, China. *A*. *niger* NH11-1 and NH3-1 are mutant strains induced by ^12^C^+6^-ion beam irradiation of strain NH01 at the Institute of Modern Physics, Academia Sinica, the Chinese Academy of Science (CCAS). *A*. *niger* NHy is a mutant strain induced by breeding *A*. *niger* NH01 with *T*. *viride* NM01 in mixed fermentation. *T*. *viride* My was obtained by breeding *A*. *niger* NH01 with *T*. *viride* NM01 in mixed fermentation. *A*. *niger* was cultured on different substrates, such as PDA slants. For chemostat cultures grown at 30°C, the cultures were stored at 4°C once adequate sporulation occurred after 4 days. *T*. *viride* was cultured on the same substrates, e.g., PDA slants, until adequate sporulation occurred after 5 days.

### Enzymes

Cellulases from a *T*. *viride* monoculture as well as *T*. *viride*/*A*. *niger* mixed culture were produced according to the method described above. Sample cellulases were harvested during the course of culturing.

### Enzyme production

#### Pre-culture and the pre-culture medium

*A*. *niger* strains were pre-cultured for 12 h until adequate biomass was obtained. This biomass was used for subsequent enzyme production. The previous step was conducted in 250-mL Erlenmeyer flasks with 50 mL of medium. The composition of the pre-culture medium (per flask) was 30 g/L sucrose, 3 g/L peptone, 5 g/L (NH_4_)_2_SO_4_, 0.4 g/L MgSO_4_ and 2 g/L KH_2_PO_4_; the medium was autoclaved at 121°C for 30 min and then aseptically inoculated with *A*. *niger* maintained on substrates such as PDA. After inoculation, the pre-culture was incubated in a shaker (HYG-XMT-C100, Shanghai Xinrui Co. Ltd., China) at 30°C and 200 rpm for 12 h. *T*. *viride* was pre-cultured for 12 h using the same medium composition as the *A*. *niger* pre-culture until adequate biomass was obtained.

#### Medium optimization for the production of enzymes

Enzyme production was performed in 250-mL Erlenmeyer flasks with 50 mL of medium, as follows: 2 mL/L Tween-80, 7.5 g/L CMC-Na, 1.4 g/L (NH_4_)_2_SO_4_, 2.0 g/L KH_2_PO_4_, 0.3 g/L CaCl_2_, 0.0016 g/L MgSO_4_.H_2_O, 0.005 g/L FeSO_4_.7H_2_O, 0.0016 g/L MnSO_4_.H_2_O, 0.0014 g/L ZnSO_4_.7H_2_O, 0.002 g/L CoCl_2_ and 1000 mL of distilled water. The prepared medium was autoclaved at 121°C for 30 min prior to use.

#### Enzyme production from monocultures of *T*. *reesei* or *A*. *niger*

Pre-cultured *T*. *reesei* or *A*. *niger* mycelium (2.5 mL) was inoculated aseptically into a flask, which was then incubated in a shaker (200 rpm) at 30°C.

#### Cellulase production from a mixed culture of *T. reesei* and *A. niger*

For mixed cultures, 4% (v/v) *T*. *viride* NMy and 1% (v/v) *A*. *niger* (NH01, NH11-1, or NH3-1), 3.1% (v/v) *T*. *viride* NMy and 1.9% (v/v) *A*. *niger* (NH01, NH11-1, or NH3-1), or 2.5% (v/v) *T*. *viride* NMy and 2.5% (v/v) *A*. *niger* (NH01, NH11-1, NH3-1) mixtures were inoculated into separate flasks. The conditions used for the mixed cultures were the same as for the monocultures.

### Methods for the analysis of enzymes

FPA activity was determined using the DNS method described by Ghose [9]. A 50-mg filter paper disc diluted in 1 mL 0.05 M citric buffer (pH 4.8) was digested by 0.5 mL of culture supernatant. The samples or blanks were incubated at 50°C for 60 min. The enzymatic reaction was terminated by adding 3 mL of DNS, followed by incubation in boiling water for 5 min. The samples were cooled in an ice bath. After sedimentation of the pulp, 20 mL of deionized water was added, and the absorbance was measured at 540 nm.

BGL activity was determined using the method described by Wu [25, 26]. A reaction mixture containing 1.5 mL of D-salicin solution and 0.5 mL of enzyme solution was incubated at 50°C for 30 min. The reaction was terminated by adding 3 mL of DNS, followed by incubating in boiling water for 5 min. The samples were cooled in an ice bath. After sedimentation of the pulp, 20 mL of deionized water was added, and the absorbance was measured at 540 nm.

EG activity was determined using the method described by Wu and Romero [25–27]. Carboxymethyl cellulose (2%) was digested using 0.5 mL of culture supernatant. The samples or blanks were incubated at 50°C for 30 min. Hydrolysis was terminated by adding 3 mL of DNS, followed by 5 min of boiling. The samples were cooled in an ice bath, 20 mL of deionized water was added, and the absorbance was measured at 540 nm.

CBH activity was determined using the method described by Wu [25, 26] and Li Zhaozhou [28]. We digested 0.5 mL of microcrystalline cellulose (1%) with 0.5 mL of culture supernatant. The samples or blanks were incubated at 30°C and 200 rpm for 20 h. Hydrolysis was terminated by adding 3 mL of DNS, followed by 5 min of boiling. The samples were cooled in an ice bath, 20 mL of deionized water was added, and the absorbance was measured at 540 nm.

### Statistical analysis

All results were analyzed with Tukey’s test (duplicate determinations, p < 0.05).

## Results and Discussion

### Survival and mutagenesis of *A*. *niger* after carbon irradiation

**Fig 1:**
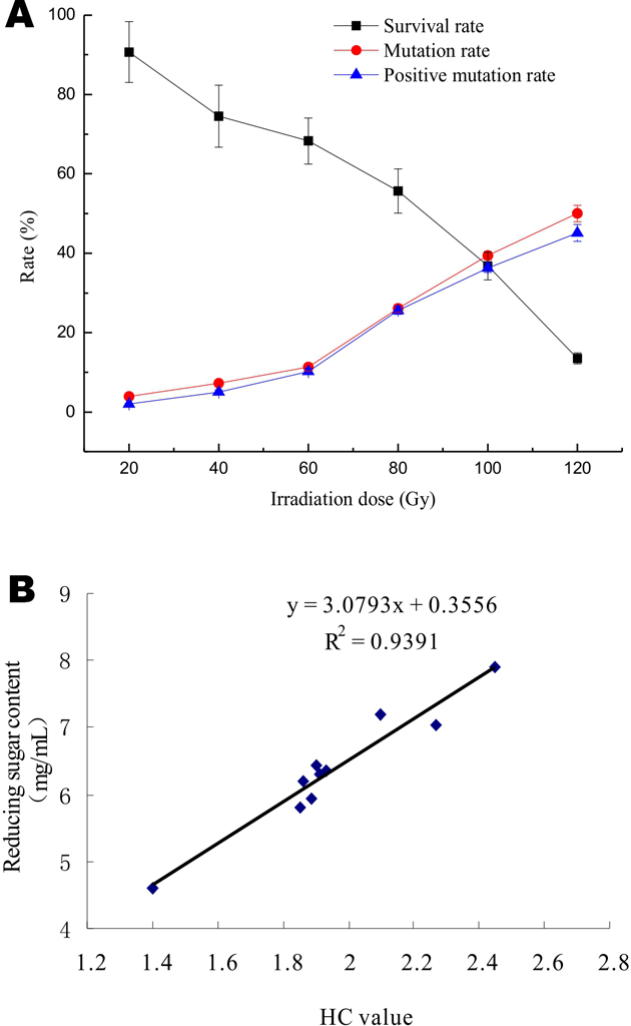
Effects of radiation dose on *Aspergillus niger*. (A) Effects of different doses of ^12^C^6+^ heavy ion radiation on survival and mutation rates. (B) Correlation between the HC value and the reducing sugar content.

*A*. *niger* strain improvement strategies, especially with regard to mutagenesis and the biological engineering of high-producing mutants, are very important to the production of metabolites during the fermentation process. In a previous study, it was reported that the lethality rate should be very high to enable the development of powerful mutations and effective mutant screening. Accordingly, heavy-ion irradiation mutagenesis has been employed to obtain high citric acid-producing *A*. *niger* mutants [29]. Investigation of survival and mutation rates in radiation-induced strains involves determining the time period required for increasing the mutation rate and improving the efficacies of synthetic components; these factors are among the most common and reliably used methods for studying the effects of radiation on microorganisms. The survival and mutant fraction values, as obtained from Eqs 1, 2 and 3, were compared with a representative set of experimental data. The *A*. *niger* strain exhibited low metabolic activity and a small HC ratio when plating was conducted after ^12^C^6+^ irradiation. However, as shown in Table 1, the ratio of mutants and the irradiation dose were still well correlated. The HC ratio and the reducing sugar content were largely changed and improved. Survival data for the *A*. *niger* colonies is shown in Fig 1A as a plot of survival against irradiation dose; ^12^C^6+^ heavy-ion irradiation (at 80 AMeV) for 20, 40, 60, 80, 100 and 120 Gy resulted in 90.7%, 14.5%, 68.3%, 55.3%, 36.5% and 13.8% survival rates, respectively. The correlation between the ratio of mutants and the irradiation dose is shown in Fig 1A; an increase in the irradiation dose led to an increase in the number of negative mutants. The rate of negative mutation for *A*. *niger* was 45%, whereas the survival rate was only 13.8% at an irradiation dose of 120 Gy. The ratio of positive mutants was only 1.04-fold greater at radiation doses of 60 Gy and 80 Gy, but the ratio of maximum positive mutants was 3.95-fold greater at a radiation dose of 120 Gy. The HC ratio and the reducing sugar content correlated well and linearly, as shown in Fig 1B (R^2^ = 0.93). To demonstrate the general applicability of our method with little modification, additional strains were included.

**Table 1:** Screening results for *Aspergillus niger* after ^12^C^6+^ irradiation.

Irradiation dose (Gy)	Colony count	Ratio range	Count of negative mutation	Count of positive mutation	Maximum ratio	Reducing sugar content (mg/mL)
20	100	0.98–1.88	2.01±0.12	2±0.13	1.88	5.95±0.03
40	100	0.85–1.93	5.03±0.53	2±0.15	1.93	6.35±0.04
60	100	0.67–1.86	10.2±0.76	1±0.21	1.86	6.20±0.49
80	100	0.45–1.82	25.6±0.98	1±0.25	1.82	6.15±0.56
100	100	0.38–2.27	36.3±1.09	2±0.31	2.27	7.03±0.98
120	100	0.36–2.45	45.1±2.11	2±0.24	2.45	7.90±0.02
0	100	1.09–1.58	**The average ratio of parent strain:** 1.40±0.03			4.61±0.08

Note: n = 3 The experiment is repeated three times

The survival rate decreased from 0.907 to 0.135 when the radiation dose was elevated from 20 to 120 Gy. A survival rate of 55–68% was noted in the case of ^12^C^6+^ irradiation, and the ratio of positive mutants decreased with an increasing radiation dose. As expected, survival depended upon the radiation dose, and an increase in irradiation strength led to a decrease in survival. At low doses, only a few mutations were induced because the damage was small and easy to repair; thus, a considerable fraction of these mutations could be effectively irradiated. In contrast, mutagenesis-induced injury was serious at high doses, with a variable range that was not easy to repair, leading to interactions between the mutations and thus reducing the surviving fraction and increasing the mutation rate. The maximum increase in the HC ratio was observed in mutants obtained at radiation doses of 100 and 120 Gy. Based on OD values, the mutant strain obtained from ^12^C^6+^ irradiation with a radiation dose of 120 Gy showed a 1.7-fold increase in HC ratio and reducing sugar content (2.45±0.04 and 7.90±0.02, respectively) compared with wild-type *A*. *niger* (1.40±0.03 and 4.61±0.08, respectively) after 3 days of fermentation, as shown in Table 1. Two mutants (*A*. *niger* NH11-1 and NH3-1) were obtained at radiation doses of 120 Gy and 100 Gy, respectively. The greatest HC ratio, 2.45, was obtained with an irradiation dose of 120 Gy. Compared with wild-type *A*. *niger* NH01, the quantity of reducing sugars obtained with the mutants was increased by 40%, reaching 7.90 mg/mL. Based on the HC ratio and high-yield reducing sugar and cellulase analogs, the estimated mutation rate (MR) was 50.02%, and the positive mutation rate (PR) was 3.95%.

### Enzyme production characteristics of *A*. *niger* after carbon irradiation

**Fig 2:**
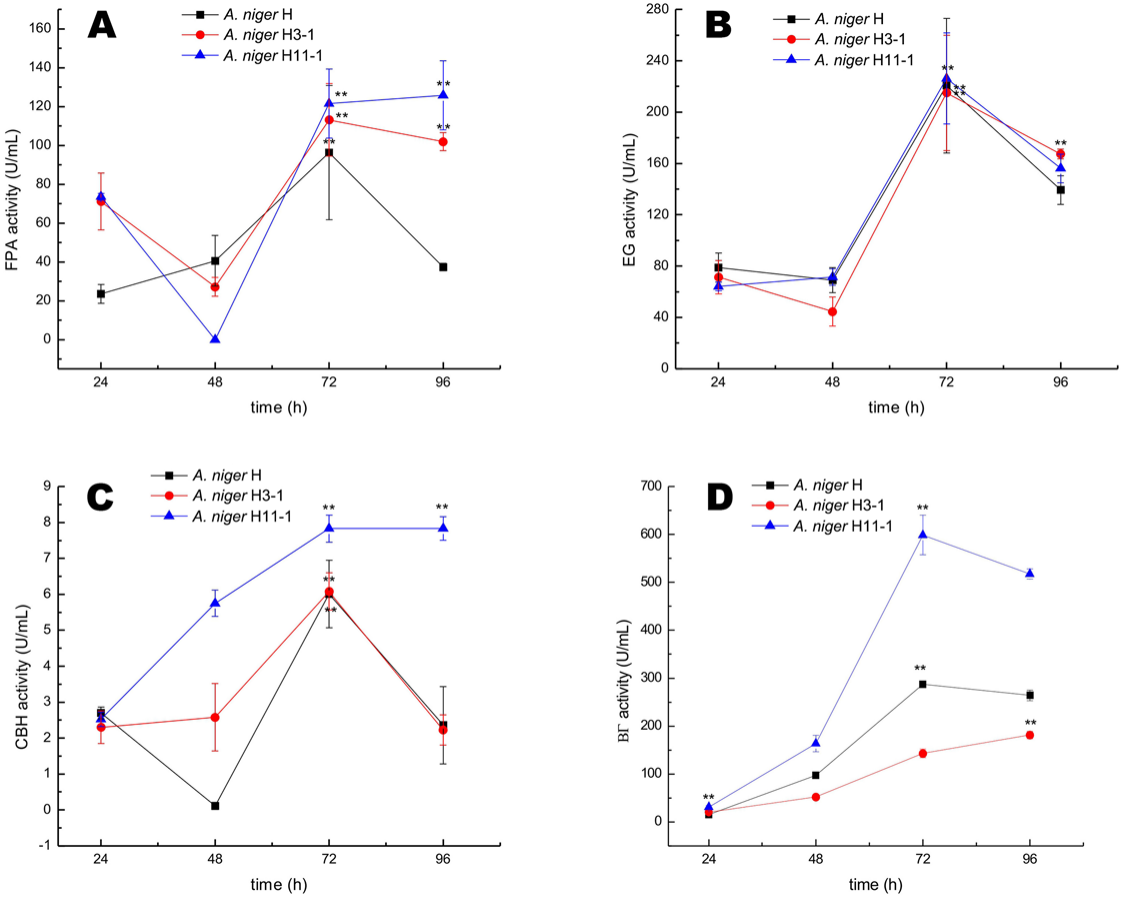
Cellulase production time courses for *Aspergillus niger* irradiated with ^12^C^6+^ heavy ions. (A) FPA activity, (B) EG activity, (C) CBH activity, (D) BGL activity.

To further compare the performance of *A*. *niger* H11-1, H3-1 and H0-1, both enzyme production and activity were studied with respect to fermentation. The experiment was conducted at the same initial pH, 30°C, and 200 rpm for 0–96 h. Fig 2 shows the time course for cellulose production and enzyme activity for *A*. *niger* H11-1, H3-1 and H0-1. Obvious differences in FPA (Fig 2A), EG (Fig 2B), CBH (Fig 2C) and BGL (Fig 2D) activities between wild-type *A*. *niger* NH-01 and the ^12^C^6+^-irradiated *A*. *niger* mutants H11-1 and H3-1 were noted, though a similar trend in the enzyme production process was observed in that maximum enzyme production was achieved at 72 h.

As noted above, after 72 h of fermentation, *A*. *niger* H11-1, NH3-1 and H0-1 demonstrated FPA (i.e., cellulase) activities of 180.44±4.68 U/mL, 131.35±12.16 U/mL and 51.39±6.55 U/mL, respectively, and EG activities of 254.28±3.74 U/mL, 164.50±11.22 U/mL and 195.36±31.79 U/mL, respectively. The CBH activities of both *A*. *niger* H11-1 and H0-1 were different from (6.71±1.22 U/mL vs. 6.01±0.28 U/mL, p < 0.05) and significantly higher than that of *A*. *niger* H3-1. The BGL activities of *A*. *niger* H11-1 and H3-1 were 626.78±0.94 U/mL and 606.44±13.09 U/mL, respectively, whereas that of *A*. *niger* H0-1 was only 464.06±14.03 U/mL. The FPA, EG and BGL activities of the high-yield mutants (H11-1 and H3-1) irradiated with ^12^C^6+^ heavy ions were higher than those of the wild-type strain H0-1 (p < 0.001). Compared with strain H01, the FPA, EG and BGL activities of mutant strain H11-1 were increased by 250.26%, 30.26% and 34.91%, respectively. Overall, ^12^C^6+^ ion irradiation induced changes in the biosynthesis of cellulase in *A*. *niger* but had no effect on the time course of the synthesis.

### Enzyme production characteristics of *A*. *niger* after mutagenesis with mixed *T*. *viride* culture

**Fig 3:**
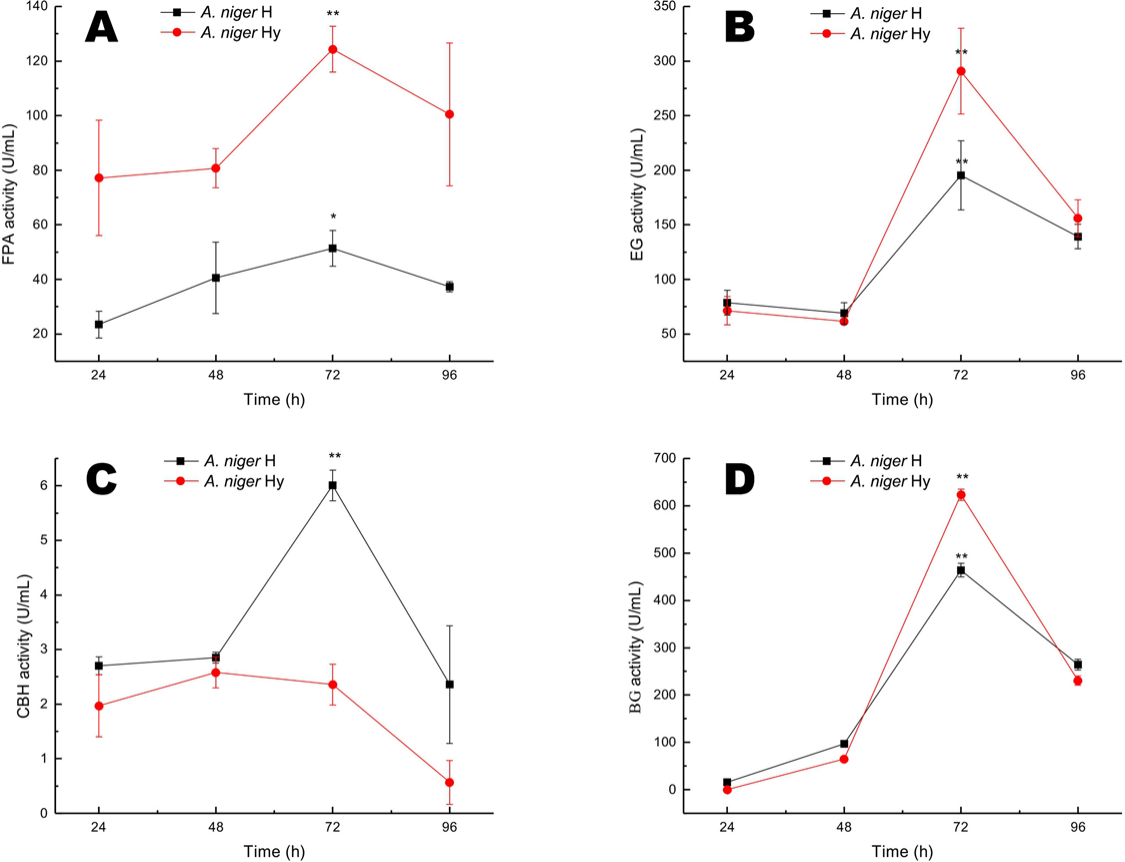
Cellulase production time courses for *Aspergillus niger* obtained from mixed culture mutation. (A) FPA activity, (B) EG activity, (C) CBH activity, (D) BGL activity.

To study the efficiency of producing high-yield mutants of *A*. *niger* induced by mixed culturing with *T*. *viride*, time courses for enzyme production and activity were studied using *A*. *niger* Hy and *A*. *niger* H0-1. The experiment was conducted under the same conditions used for *A*. *niger* after carbon irradiation. Fig 3 reveals a similar trend in the enzyme production process of *A*. *niger* wild-type strain H0-1 and the mutagenized Hy strains induced by mixed *T*. *viride* culturing; maximum enzyme production was achieved at 72 h. During enzyme production, the FPA activity exhibited by the mutant strain Hy was significantly higher than that of wild-type strain H0-1 at 24, 48, 72 and 96 h (Fig 3A); however, its CBH activity was significantly lower than that of wild-type strain H0-1 (Fig 3C). The EG and BGL activities of mutant *A*. *niger* Hy were significantly higher than that of wild-type strain H0-1 only after 72 h of fermentation (Fig 3B, Fig 3D), but the enzyme production processes in the heavy-ion mutant strain, the mixed culture mutant strain, and the wild-type strain followed a similar trend, with maximum enzyme production achieved at 72 h.

Compared with wild-type strain H0-1, the *A*. *niger* mutant induced by mixed *Trichoderma* culturing exhibited 250.26%, 30.26% and 34.91% (p < 0.001) higher FPA, EG and BGL activities, respectively; however, the CBH activity of mixed culture mutant strain Hy was reduced by 154.66% (p < 0.001). Mixed-culturing mutations induced changes in the biosynthesis of cellulase in *A*. *niger* but had no effect on the time course of the synthesis.

### Improvement in cellulase production via mixed culture

**Fig 4:**
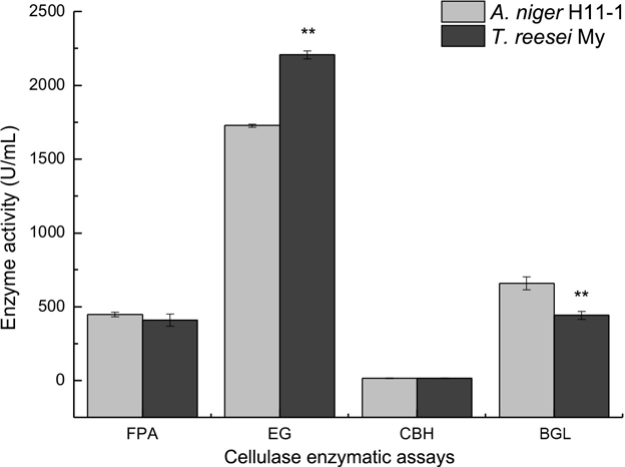
Final FPA, EG, CBH and BGL enzyme activities were determined directly from fermented broth obtained from monocultures at various time points (*T*. *reesei* My or *A*. *niger* H11-1) grown in a volume of 50 mL in 250-mL conical flasks and incubated with shaking at 30°C for 96 h at 200 rpm. The inoculum volume of *T*. *reesei* or *A*. *niger* for monocultures was 2.5 mL.

Improved compositions of cellulolytic enzymes from *A*. *niger* H11-1, *T*. *viride* My and a mixed culture system of *A*. *niger* and *T*. *reesei* were used to produce cellulase with excellent catalytic and compositional characteristics. Fig 4 shows the FPA, EG, CBH and BGL activities from *A*. *niger* H11-1 and *T*. *viride* after 72 h of fermentation. The FPA and BGL activities of *A*. *niger* H11-1 were higher than those of *T*. *viride* My, whereas the EG activity of *T*. *viride* My was significantly higher than that of *A*. *niger* H11-1.

**Fig 5:**
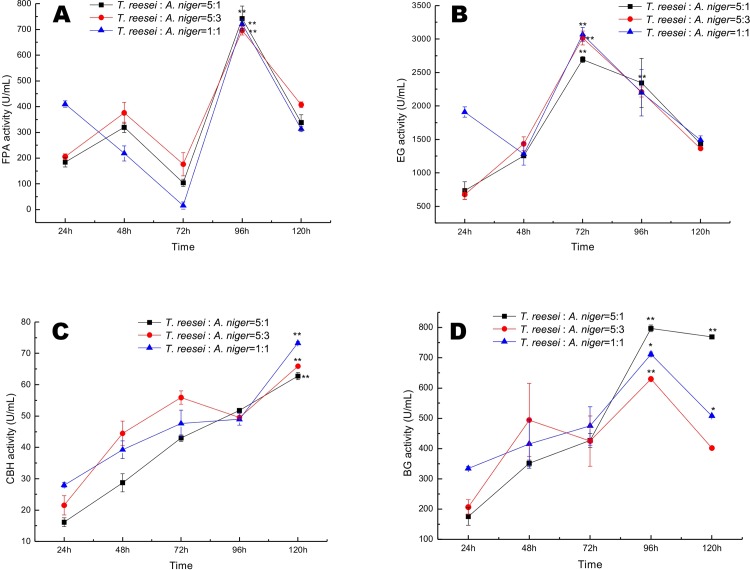
FPA (A), EG (B), CBH (C) and BGL (D) enzyme activities were determined directly from fermented broth obtained at various time points from mixed cultures of *T*. *reesei* My and *A*. *niger* H11-1 inoculated at different ratios (5:1, 5:3 and 1:1) and grown in a volume of 50 mL in 250-mL conical flasks with shaking at 30°C for 96 h at 200 rpm.

**Fig 6:**
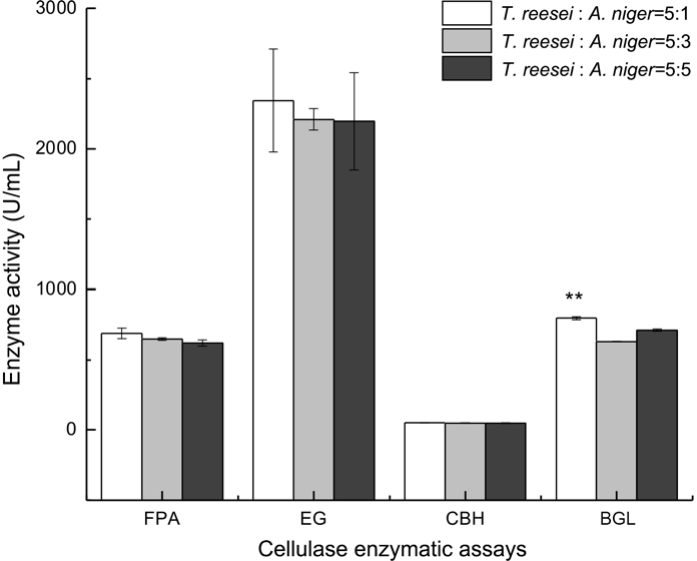
Final FPA, EG, CBH and BGL enzyme activities were determined directly from fermented broth obtained at various time points from mixed cultures of *T*. *reesei* My and *A*. *niger* H11-1 inoculated at different ratios (5:1, 5:3 and 1:1) and grown in a volume of 50 mL in 250-mL conical flasks with shaking at 30°C for 96 h at 200 rpm.

Mixed culture systems were established with inoculum ratios of *T*. *reesei* to *A*. *niger* of 5:1, 5:3 and 1:1. Data for the cellulolytic enzyme yield as a function of time (5 days) for each inoculum ratio are presented in Fig 5. The mixed culture systems reached their maximum FPA and BGL activities at 96 h, which was the time of cellulase harvest. After 96 h, the FPA and BGL activities of the mixed cultures decreased (Fig 5), but EG activity in the mixed cultures reached a maximum at 72 h. At 96 h, the FPA and BGL activities of the 5:1 inoculum ratio at 96 h were increased by 6.8% and 3.1%, respectively, compared with those of the 5:3 system and 26.71% and 11.94%, respectively, compared with those of the 1:1 system. The EG activity of the 5:1 system at 96 h was increased by 6.05% and 6.67% compared with that of that of the 5:3 and 1:1 systems, respectively. A mixed culture system was successfully established with *T*. *reesei* and *A*. *niger* at a ratio of 5:1 at 96 h with simultaneous inoculation (Fig 6). We found similar results as Fang et al., in that the inoculum ratio of *T*. *reesei* to *A*. *niger* was 5:1, but Fang et al. established a mixed culture system by delaying *A*. *niger* inoculation by 48 h, altering the amount of time (7 days) in monoculture and growing the mixed cultures in SECS culture medium.

**Fig 7:**
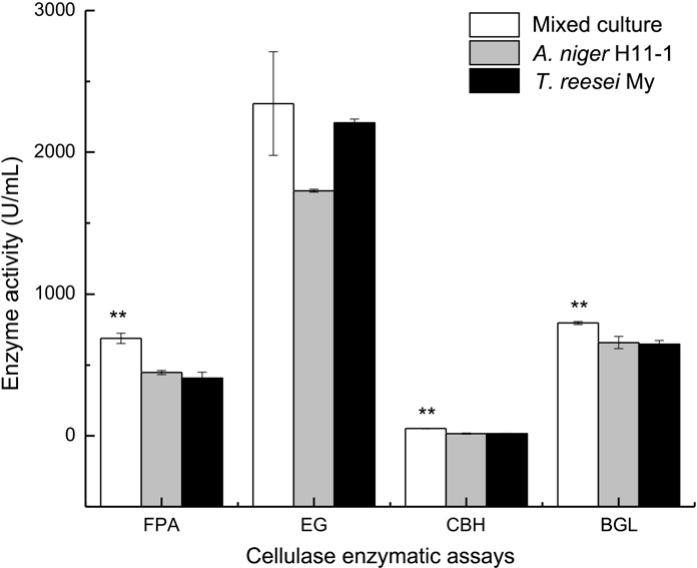
Final FPA, EG, CBH and BGL enzyme activities were determined directly from fermented broth obtained at various time points from monocultures (*T*. *reesei* My or *A*. *niger* H11-1) and mixed cultures (*T*. *reesei* My and *A*. *niger* H11-1) grown in a volume of 50 mL in 250-mL conical flasks with shaking at 30°C for 96 h at 200 rpm. The inoculum volume of *T*. *reesei* or *A*. *niger* was 2.5 mL for monocultures; the inoculum ratio of *T*. *reesei* to *A*. *niger* was 5:1 for the mixed cultures, with simultaneous inoculation.

The FPA, EG, CBH and BGL activities in both monocultures and mixed cultures after 96 h of fermentation are shown in Fig 7. With respect to the detection and analysis of relationships among these data, some soluble cellobiose and cellodextrins may still have existed in the medium, inducing BGL secretion from *A*. *niger* after cellulose was exhausted. The BGL activity of the mixed culture was still increased after 72 h, and at 96 h, the FPA and BGL activities of the mixed culture were 689.00±36.89 and 797.15±11.14 U/mL, respectively. These activities were significantly higher than those of the monocultures, which were 408.70±40.85 and 646.98±27.23 U/mL, respectively, for *T*. *reesei* and 447.29±15.13 and 658.89±43.12 U/mL, respectively, for *A*. *niger*. The EG activity of the mixed culture was 2342.81±36.62 U/mL, which was significantly higher than that of the monocultures, at 2206.57±27.23 U/mL for *T*. *reesei* and 1727.62±10.59 U/mL for *A*. *niger*. This result may have occurred because the *T*. *reesei/A*. *niger* mixed culture led to enhanced cellulolytic enzyme production and suggests that the two different microorganisms interacted synergistically with the substrate by secreting a complete enzyme complex [30]. Because the BGL-deficient *T*. *reesei* cellulase system caused the accumulation of cellobiose, BGL secretion was strongly induced from *A*. *niger*. Under conditions in which the two strains utilize the substrates efficiently, BGL catalysis of the accumulated cellobiose is attributable to cellobiose induction from *A*. *niger*. A strong inducer of cellulase production, gentiobiose is capable of inducing the de-glycosylation of substrates caused by the synergistic interaction of the cellulase enzyme complex in mixed cultures [31], thus enhancing cellulase production from the mixed culture system. The above phenomenon may explain why FPA, EG and BGL were increased in a mixed culture of *T*. *reesei* and *A*. *niger*.

## Conclusions

The mixed culture of microorganisms can be used to produce high-efficiency mixed cultures. *A*. *niger* is good for the production of BGL, but its EGs production capacity is low. *T*. *viride* has a strong ability to produce endoglucanases. Thus, mutagenesis together with mixed *T*. *viride* makes sense. *A*. *niger* was screened for a high EG substrate affinity, and EGs and BGLs were obtained in strains with higher mutation rates. In addition, mutant strains screened via hybrid mutation have good advantages in mixed fermentation. The FPA, EG, CBH and BGL activities of *A*. *niger* H11-1 induced by ^12^C^6+^ irradiation were higher than those of *A*. *niger* Hy, the mutations of which were induced by mixed *Trichoderma* culturing, after 96 h of fermentation. Of note, the exonuclease and *β*-glucosidase enzymes produced by *A*. *niger* H11-1 have clear advantages compared with those from the *A*. *niger* Hy strains. The mutant strain was obtained by combining targeted screening to show overall improved performance, with a greater complexity and diversity of mutants. Moreover, this strategy is very effective for enhancing cellulase production in a mixed culture of mutant *T*. *viride* and *A*. *niger*.
